# Real‐World Outcomes of Hypomethylating Agents for Higher‐Risk Myelodysplastic Syndromes: Report From the Nationwide Multicenter Thai MDS Study

**DOI:** 10.1155/ah/2732324

**Published:** 2026-09-15

**Authors:** Theerin Lanamtieng, Chantana Polprasert, Pimjai Niparuck, Phatsorn Choksomnuk, Thanawat Rattanathammethee, Wasithep Limvorapitak, Kannadit Prayongratana, Rattapan Lamool, Archrob Khuhapinant, Weerapat Owattanapanich, Pisa Phiphitaporn, Kanchana Chansung, Suporn Chuncharunee

**Affiliations:** ^1^ Division of Hematology, Department of Medicine, Faculty of Medicine, Khon Kaen University, Khon Kaen, Thailand, kku.ac.th; ^2^ Division of Hematology, Department of Medicine and Center of Excellence in Translational Hematology, Faculty of Medicine, Chulalongkorn University, Bangkok, Thailand, chula.ac.th; ^3^ Division of Hematology, Department of Medicine, Faculty of Medicine, Ramathibodi Hospital, Mahidol University, Bangkok, Thailand, mahidol.ac.th; ^4^ Division of Hematology, Department of Internal Medicine, Faculty of Medicine, Chiang Mai University, Chiang Mai, Thailand, cmu.ac.th; ^5^ Division of Hematology, Department of Medicine, Thammasat University, Pathumthani, Thailand, tu.ac.th; ^6^ Division of Hematology, Department of Internal Medicine, Phramongkutklao Hospital and College of Medicine, Bangkok, Thailand, pmk.ac.th; ^7^ Division of Hematology, Department of Medicine, Faculty of Medicine, Siriraj Hospital, Mahidol University, Bangkok, Thailand, mahidol.ac.th

**Keywords:** clinical outcome, hypomethylating agents, myelodysplastic syndrome, survival

## Abstract

Hypomethylating agents (HMAs) are the standard of care for higher‐risk myelodysplastic syndromes (MDSs); however, their accessibility remains limited. This retrospective multicenter cohort study aimed to evaluate the real‐world overall survival (OS) of HMAs compared to best‐available treatments (BATs) in newly diagnosed higher‐risk MDS in Thailand. Higher‐risk MDS was defined by a Revised International Prognostic Scoring System (IPSS‐R) score > 3.5. Data were extracted from the MDS registry of multicenter hospitals in Thailand from 2013 to 2023. Patients who underwent hematopoietic stem cell transplantation were excluded. A total of 155 patients were included, with a median age of 70 years and a male predominance. The median follow‐up time was 36 months. Of these, 86 patients (55.5%) received BATs, while 69 patients (44.5%) were treated with HMAs. HMAs were found to significantly improve OS compared to BATs (log‐rank test: *p* = 0.03), with a median OS of 15 months versus 6 months, respectively. Multivariable Cox regression analysis revealed that HMAs were associated with a decreased risk of death (hazard ratio: 0.58, 95% CI, 0.38–0.89, *p* = 0.01), while age and high‐ and very high‐risk IPSS‐R were identified as poor prognostic factors. This study underscores the real‐world effectiveness of HMAs for higher‐risk MDS in Thailand.

## 1. Introduction

Myelodysplastic syndromes (MDSs) are a heterogeneous group of clonal hematopoietic disorders characterized by cytopenias, dysplastic bone marrow morphology, and a variable risk of progression to acute myeloid leukemia (AML). Patients with higher‐risk disease, as defined by the Revised International Prognostic Scoring System (IPSS‐R), face particularly poor outcomes, with median overall survival (OS) often less than 2 years [1]. Allogeneic hematopoietic stem cell transplantation (HSCT) remains the only curative therapy, but most patients are ineligible due to advanced age, comorbidities, or a lack of donor availability [2].

For nontransplant candidates, hypomethylating agents (HMAs) such as azacitidine and decitabine are the standard of care [3]. The landmark AZA‐001 trial [4] demonstrated that azacitidine significantly prolonged OS compared with conventional care regimens, reporting a median OS of 24.5 months versus 15 months, thereby establishing HMAs as the preferred treatment for higher‐risk MDS. However, subsequent real‐world studies have reported more modest outcomes, with median OS typically ranging between 12 and 18 months, reflecting broader patient populations and treatment challenges [5–11].

In Thailand, where healthcare resource limitations and restricted drug accessibility remain significant challenges, the real‐world outcomes of HMA treatment in higher‐risk MDS are poorly documented. To address this gap, we conducted a nationwide, multicenter retrospective study to evaluate the effectiveness of HMAs compared with best‐available treatments (BATs) and to identify prognostic factors associated with survival in Thai patients with higher‐risk MDS.

## 2. Materials and Methods

### 2.1. Study Design and Population

We performed a retrospective cohort study using data from the Thai Nationwide MDS Registry, comprising multiple university hospitals across Thailand. Patients diagnosed with higher‐risk MDS between January 2013 and December 2023 were eligible if their IPSS‐R score was greater than 3.5. Patients who underwent HSCT were excluded.

### 2.2. Study Sites

This study was conducted across seven major university hospitals and tertiary care centers in Thailand, reflecting nationwide representation. Participating institutions included Srinagarind Hospital at Khon Kaen University in Khon Kaen, King Chulalongkorn Memorial Hospital at Chulalongkorn University in Bangkok, and Ramathibodi Hospital at Mahidol University in Bangkok. Additional sites were Maharaj Nakorn Chiang Mai Hospital at Chiang Mai University in Chiang Mai, Thammasat University Hospital at Thammasat University in Pathum Thani, Phramongkutklao Hospital and College of Medicine in Bangkok, and Siriraj Hospital at Mahidol University in Bangkok.

### 2.3. Definitions

Higher‐risk MDS was defined as an IPSS‐R score > 3.5 (intermediate, high, and very high categories). Patients were categorized into two treatment groups: the HMA group (treated with azacitidine or decitabine) and the BAT group (receiving supportive care, growth factors, low‐dose cytarabine, or immunosuppressive therapy).

### 2.4. Outcomes

The primary outcome was OS, defined as the time from diagnosis to death from any cause or to the last follow‐up. Secondary outcomes included prognostic factors associated with OS, focusing on age, IPSS‐R risk categories, and cytogenetic abnormalities.

### 2.5. Statistical Analysis

Baseline characteristics were summarized descriptively. Survival was analyzed using Kaplan–Meier curves, with comparisons made by log‐rank tests. Univariable and multivariable Cox proportional hazards regression models were performed to identify independent prognostic factors for OS, including treatment modality, age, and IPSS‐R category. Hazard ratios (HRs) and 95% confidence intervals (CIs) were reported.

To further reduce potential treatment selection bias, a propensity score matching analysis was performed as a sensitivity analysis. Propensity scores for receipt of HMAs were estimated using a logistic regression model including age, sex, and IPSS‐R risk category. Patients receiving HMAs were matched 1:1 with patients not receiving HMAs using nearest‐neighbor matching without replacement. Covariate balance before and after matching was assessed using standardized mean differences and visualized with a Love plot. OS in the matched cohort was compared using the Kaplan–Meier method and the log‐rank test.

Subgroup analyses were performed to evaluate the association between HMA treatment and OS across clinically relevant patient subgroups. Patients were stratified according to age (< 65 vs. ≥ 65 years), IPSS‐R risk category (intermediate, high, and very high), and IPSS‐R cytogenetic risk group (good/intermediate vs. poor/very poor). Within each subgroup, OS was compared using univariable Cox proportional hazards regression. HRs with corresponding 95% CI were estimated and presented as a forest plot.

All statistical analyses were conducted using *R* Statistical Software (v 4.3.2; *R* Core Team, 2023). The study was reviewed and approved by the Khon Kaen University Ethics Committee for Human Research in accordance with the Declaration of Helsinki and the ICH Good Clinical Practice guidelines (HE671179). The approval was granted on March 18, 2024. According to the approval from the ethics committee, informed consent was waived because the study involved analysis of existing data without any prospective data collection or direct interaction with participants.

## 3. Results

A total of 155 patients with newly diagnosed higher‐risk MDS were included in the study. The median follow‐up time for the overall cohort was 36 months (interquartile range [IQR]: 17–69). The median age at diagnosis was 70 years (range, 38–88), and 94 patients (60.6%) were male. Of the cohort, 69 patients (44.5%) received HMAs, primarily azacitidine (98.1%), while 86 patients (55.5%) received BATs (Table 1). Patients treated with HMAs were, on average, younger and had higher baseline platelet counts compared with those receiving BATs.

**TABLE 1:** Baseline characteristics.

	Best‐available treatments *N* = 86	Hypomethylating agents *N* = 69	*p* value
Male (%)	51 (59.3)	43 (62.3)	0.83
Age (median [IQR])	71.50 [63.00, 80.00]	67.00 [59.00, 73.00]	0.01
Hemoglobin (g/dL) (median [IQR])	7.15 [6.50, 8.30]	7.40 [6.60, 8.90]	0.29
Absolute neutrophil count (× 10^3^/μL) (median [IQR])	1.06 [0.46, 1.8]	1.44 [0.5, 1.97]	0.16
Platelet (× 10^3^/μL) (median [IQR])	59 [35, 103]	94 [48, 138]	0.01
Bone marrow blast percentage (median [IQR])	7.70 [3.00, 12.00]	10.00 [5.00, 14.00]	0.05
IPSS‐R cytogenetic risk group (%)			0.65
Very good	0 (0)	0 (0)	
Good	37 (43.0)	31 (44.9)	
Intermediate	18 (20.9)	16 (23.2)	
Poor	8 (9.3)	9 (13.0)	
Very poor	23 (26.7)	13 (18.8)	
IPSS‐R risk (%)			0.71
Intermediate	14 (16.3)	10 (14.5)	
High	45 (52.3)	33 (47.8)	
Very high	27 (31.4)	26 (37.7)	

*Note:* IQR: interquartile range, IPSS‐R: Revised International Prognostic Scoring System.

Cytogenetic analysis showed that 43.9% of patients had a normal karyotype, 23.2% had complex chromosomal abnormalities, 14.8% had Monosomy 7, 9.7% had Trisomy 8, and 6.5% had Monosomy 5. Chromosomal abnormalities for the entire cohort and for each treatment group are presented in Table 2. Genetic profiling was available for only 10.9% of the total cohort (17/155). Among these, *ASXL1* was the most commonly mutated gene.

**TABLE 2:** Cytogenetic profile of patients at diagnosis.

Chromosome	All cohort	Best‐available treatments	Hypomethylating agents
Normal chromosome	68 (43.9%)	30 (43.5%)	38 (44.2%)
Complex chromosome	36 (23.2%)	13 (18.8%)	23 (26.7%)
Monosomy 7	23 (14.8%)	10 (14.5%)	13 (15.1%)
Trisomy 8	15 (9.7%)	5 (7.2%)	10 (11.8%)
Monosomy 5	10 (6.5%)	4 (5.8%)	7 (8.1%)
Deletion 5q	7 (4.5%)	3 (4.3%)	6 (7%)
Monosomy 20	7 (4.5%)	3 (4.3%)	4 (4.7%)
Translocations involving Chromosome 3	6 (3.9%)	3 (4.3%)	4 (4.7%)
Monosomy 18	6 (3.9%)	3 (4.3%)	4 (4.7%)
Trisomy 21	6 (3.9%)	3 (4.3%)	4 (4.7%)
Deletion 13	5 (3.2%)	2 (2.9%)	4 (4.7%)
Deletion 17	5 (3.2%)	2 (2.9%)	3 (3.5%)
Deletion 19	5 (3.2%)	2 (2.9%)	3 (3.5%)
Isochromosome 17q	5 (3.2%)	1 (1.4%)	3 (3.5%)
Deletion 7	4 (2.6%)	1 (1.4%)	2 (2.3%)
Deletion 9	4 (2.6%)	1 (1.4%)	2 (2.3%)
Monosomy 9	4 (2.6%)	1 (1.4%)	2 (2.3%)
Deletion 20	3 (1.9%)	0 (0%)	2 (2.3%)

Among patients receiving BATs, 44% were treated with blood transfusions alone, 16% were treated with growth factors alone, 13% received blood transfusions in combination with growth factors, 1% received immunosuppressive drugs, 1% received chemotherapy, and 24% received palliative care.

The median number of HMA cycles administered was 5 (IQR: 2–10). Response rates according to the International Working Group (IWG) 2006 criteria were available for 55.1% (38/69) of patients in the HMA group. Among these patients, 5.3% achieved partial response (PR), 28.9% had progressive disease (PD), 52.6% showed hematologic improvement (HI), 7.9% had stable disease (SD), and 5.3% achieved marrow complete remission (mCR). The overall response rate (ORR; defined as the proportion of patients achieving CR, PR, mCR, or HI) was 63.2%.

**FIGURE 1:**
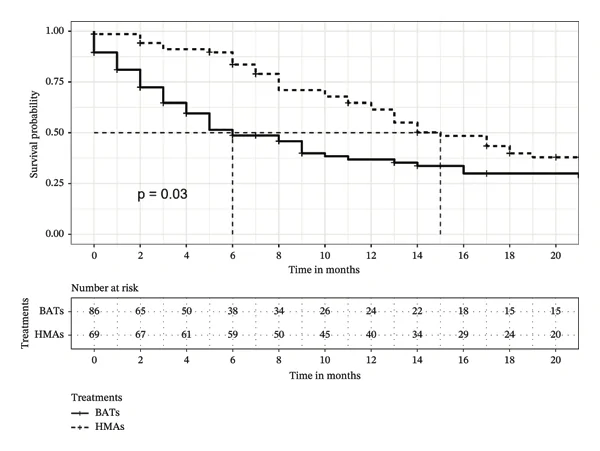
Kaplan–Meier curves for the entire cohort (BATs: best‐available treatments, HMAs: hypomethylating agents).

Patients treated with HMAs had significantly longer survival compared with those receiving BATs. Median OS in the HMA group was 15 months, whereas in the BAT group it was only 6 months, with the difference reaching statistical significance (log‐rank *p* = 0.03, with an HR of 0.66 (95% CI, 0.45–0.97)) (Figure 1). In multivariable Cox regression analysis, HMA therapy was independently associated with improved OS, with an HR of 0.58 (95% CI, 0.38–0.89, *p* = 0.01) (Table 3).

**TABLE 3:** Hazard ratio from univariable and multivariable cox regression analysis.

Variable	Hazard ratio (95% confidence interval)	*p* value
*Univariable Cox regression analysis*		
Hypomethylating agents	0.66 (0.45–0.97)	0.03
Age	1.02 (1.01–1.03)	0.01
Gender (male)	1.32 (0.89–1.96)	0.17
IPSS‐R category (high)	4.75 (2.24–10.07)	< 0.01
IPSS‐R category (very high)	6.94 (3.21–14.97)	< 0.01

*Multivariable Cox regression analysis*		
Hypomethylating agents	0.58 (0.38–0.89)	0.01
Age	1.02 (1.01–1.04)	0.03
Gender (male)	1.15 (0.77–1.72)	0.50
IPSS‐R category (high)	4.75 (2.24–10.05)	< 0.01
IPSS‐R category (very high)	8.71 (3.95–19.20)	< 0.01

In addition to treatment modality, age and IPSS‐R risk group were important prognostic variables. Older age was associated with increased mortality (HR 1.02 per year, 95% CI, 1.01–1.04, *p* = 0.03). Patients in the high‐ and very high‐risk IPSS‐R category experienced significantly poorer OS compared with those in the intermediate categories (HR 4.75, 95% CI, 2.24–10, *p* < 0.01 and HR 8.71, 95% CI, 3.95–19.20, *p* < 0.01, respectively) (Table 3).

**FIGURE 2:**
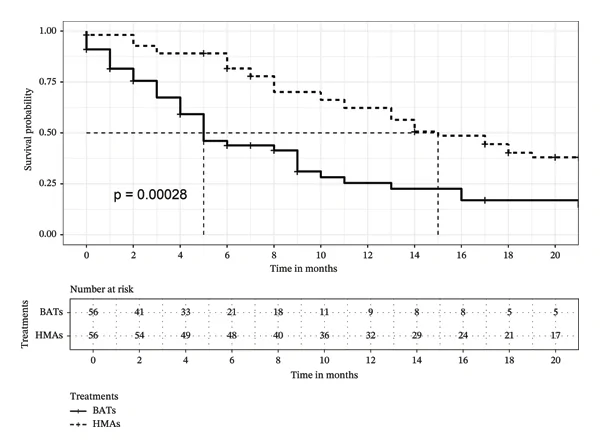
Kaplan–Meier curves for the matched cohort (BATs: best‐available treatments, HMAs: hypomethylating agents).

A propensity score matching analysis was performed to minimize potential confounding due to baseline differences between treatment groups. Patients were matched 1:1 according to age, sex, and IPSS‐R risk category. After matching, all covariates included in the propensity score model achieved standardized mean differences < 0.10, indicating good covariate balance (Supporting Figure 1 and Supporting Table 1). Kaplan–Meier analysis of the matched cohort demonstrated that patients treated with HMAs had significantly longer OS (log‐rank *p* < 0.01) (Figure 2). These findings were consistent with the results of the multivariable Cox regression analysis, supporting the robustness of the observed survival benefit associated with HMA treatment.

**FIGURE 3:**
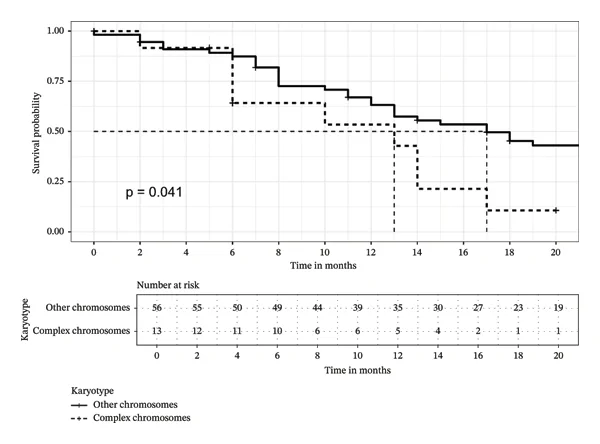
Kaplan–Meier curves for patients receiving HMAs (HMAs: hypomethylating agents).

Within the subgroup of patients treated with HMAs, complex cytogenetic abnormalities exhibited inferior outcomes compared with patients with noncomplex karyotypes (log‐rank *p* = 0.04; HR 2.19, 95% CI, 1.03–4.66) (Figure 3), while Monosomy 7 (log‐rank *p* = 0.18; HR 1.74, 95% CI, 0.78–3.86) and Trisomy 8 (log‐rank *p* = 0.06; HR 2.48, 95% CI, 0.95–6.45) did not show a statistically significant association with survival.

**FIGURE 4:**
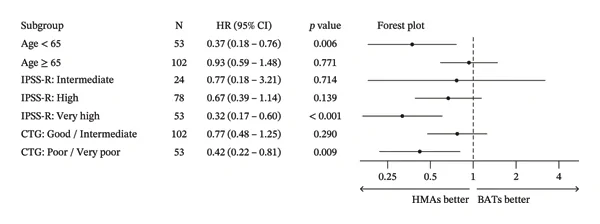
Forest plot showing hazard ratios for overall survival comparing best‐available treatment (BAT)–treated and hypomethylating agent (HMA)–treated patients across subgroups (IPSS‐R: Revised International Prognostic Scoring System, CTG: cytogenetic group).

A survival benefit associated with HMA treatment was observed among patients aged < 65 years (*n* = 53), those with IPSS‐R very high‐risk disease (*n* = 53), and patients with poor or very poor cytogenetic risk (*n* = 53). In contrast, no significant survival benefit was observed among patients aged ≥ 65 years, those with IPSS‐R intermediate or high‐risk disease, and patients with good or intermediate cytogenetic risk (Figure 4).

## 4. Discussion

This nationwide, multicenter study provides real‐world evidence supporting the effectiveness of HMAs in Thai patients with higher‐risk MDS. Treatment with HMAs was associated with significantly longer survival compared with BATs, with a median OS of 15 months versus 6 months. These results align with global experience, although survival outcomes were shorter than those reported in the AZA‐001 trial (24.5 months) [4]. The discrepancy likely reflects broader eligibility in real‐world cohorts, older patient populations, and variability in supportive care, as shown in other registry‐based studies where median OS ranged from 12 to 18 months [5].

In this study, 44.5% of the cohort received HMAs, which is comparable to that of a large‐scale observational study conducted by the Japan Adult Leukemia Study Group [11]. When comparing ORR, our study demonstrated an ORR of 63.2%, which is comparable to the outcomes reported in a large real‐world cohort of higher‐risk MDS patients in the United States (62.8%) [7]. However, IWG response was available for only 55%, so CR may have been underreported. Therefore, the absence of CR in our study should be interpreted with caution.

Access to HMAs in Thailand has been limited by reimbursement policies. During the study period, HMA reimbursement was primarily available through the Civil Servant Medical Benefit Scheme, whereas patients covered by the Universal Coverage Scheme or Social Security Scheme often had limited access because of the high treatment cost. Encouragingly, reimbursement policies are evolving. The real‐world outcomes presented in this study provide locally relevant evidence supporting the effectiveness of HMAs in Thai patients, which may help inform healthcare policy and facilitate broader reimbursement.

The median OS in the BAT group was shorter than that reported in several previous studies. This may be explained by the composition of the BAT group in our cohort, in which most patients received supportive or palliative management, including blood transfusions, growth factors, or palliative care, whereas only a small proportion received disease‐modifying therapies such as immunosuppressive therapy or chemotherapy.

We confirmed the prognostic relevance of IPSS‐R in real‐world practice, with high‐ and very high‐risk disease emerging as a strong predictor of poor OS. This is consistent with prior validation studies demonstrating that IPSS‐R stratification maintains prognostic utility across different treatment settings [9]. Patients in the very high‐risk group typically present with adverse cytogenetic features, severe cytopenias, and rapid disease progression, which translate into limited benefit even from HMA therapy. Novel therapeutic approaches or early clinical trial enrollment should be prioritized for this subgroup.

Another key finding was the poor outcome of patients with complex karyotypes despite HMA treatment. This observation mirrors prior studies linking complex cytogenetics with TP53 mutations, which confer intrinsic resistance to HMAs and drive poor survival [12, 13]. Recent investigational therapies, including the p53 reactivator APR‐246 (eprenetapopt) and the anti‐CD47 antibody magrolimab, were previously explored in this high‐risk subgroup [14]. However, both agents have shown limited efficacy, making them unavailable and leaving patients with very few treatment options beyond standard therapies.

Age was independently associated with worse OS, consistent with global registries showing that advanced age contributes both to disease biology and to reduced tolerance of treatment [8, 15]. This underscores the importance of balancing treatment efficacy with supportive care optimization in elderly MDS patients.

Our subgroup analyses suggest that the survival benefit may be more apparent in patients with biologically higher‐risk disease. A benefit was also observed among patients younger than 65 years. However, no statistically significant survival advantage was detected in the remaining subgroups. The landmark AZA‐001 trial demonstrated that the OS benefit of azacitidine was generally consistent across clinically relevant subgroups. In contrast, our study identified significant associations only in selected higher‐risk subgroups. This discrepancy is likely explained by the smaller sample size of our cohort compared with the AZA‐001 trial and the limited statistical power available for subgroup analyses.

This study has several limitations. As with all retrospective studies, residual confounding cannot be completely excluded despite multivariable adjustment and propensity score matching. OS was calculated from the time of diagnosis, whereas the exact date of HMA initiation was not available in the registry. Consequently, we were unable to perform a landmark analysis, and the possibility of immortal time bias cannot be completely excluded.

Molecular data, including TP53 and other gene mutations, were available for only 10.9% of the cohort due to limited resources and restricted access to genetic sequencing. This prevented more detailed analyses of genetic prognostic factors and their potential impact on treatment response and OS. Consequently, the Molecular International Prognostic Scoring System (IPSS‐M) [16], which is currently considered the standard prognostic model for MDS, could not be applied. Therefore, we used IPSS‐R, which was the previous standard prognostic system during the study period and could be consistently applied across the study cohort.

In addition, the registry did not capture detailed treatment‐related information, including HMA treatment schedule, dose intensity, reasons for treatment discontinuation, treatment‐related toxicities, infection‐related mortality, or time to response. Consequently, we were unable to evaluate treatment adherence, treatment modifications, or the safety profile of HMAs. Finally, treatment heterogeneity and differences in supportive care practices across centers may have influenced outcomes.

This study has important strengths. This is a real‐world, multicenter dataset that represents the actual use of HMAs in routine clinical practice across multiple regions of Thailand. As such, the findings are generalizable to the broader Thai patient population and provide valuable insights into real‐life treatment outcomes, including the benefits and limitations of HMAs outside of clinical trials.

This nationwide, multicenter study demonstrates that HMAs significantly improve survival compared with BATs in Thai patients with higher‐risk MDS. Older age and high‐ and very high‐risk IPSS‐R were identified as poor prognostic factors, confirming their clinical relevance in real‐world practice. Improving access to HMAs for patients with higher‐risk MDS is critical for achieving better outcomes in this challenging disease.

## Author Contributions

Theerin Lanamtieng: data curation, resources, conceptualization, writing–original draft, visualization, investigation, methodology, and formal analysis.

Chantana Polprasert: data curation, supervision, writing–review and editing, funding acquisition, methodology, formal analysis, and conceptualization.

Pimjai Niparuck: data curation, resources, and writing–review and editing.

Phatsorn Choksomnuk: data curation and resources.

Thanawat Rattanathammethee: data curation, resources, validation, and writing–review and editing.

Wasithep Limvorapitak: data curation, resources, and writing–review and editing.

Kannadit Prayongratana: data curation, resources, and writing–review and editing.

Rattapan Lamool: data curation, resources, and writing–review and editing.

Archrob Khuhapinant: data curation, resources, and writing–review and editing.

Weerapat Owattanapanich: data curation, resources, and writing–review and editing.

Pisa Phiphitaporn: data curation and resources.

Kanchana Chansung: data curation, resources, project administration, and writing–review and editing.

Suporn Chuncharunee: data curation, project administration, supervision, and writing–review and editing.

## Funding

This study was supported by the Thai Society of Hematology.

## Disclosure

All authors have read and approved the final version of the manuscript. Theerin Lanamtieng had full access to all of the data in this study and takes complete responsibility for the integrity of the data and the accuracy of the data analysis.

## Ethics Statement

The study was reviewed and approved by the Khon Kaen University Ethics Committee for Human Research in accordance with the Declaration of Helsinki and the ICH Good Clinical Practice guidelines (grant number: HE671179). The approval was granted on March 18, 2024.

According to the approval from the ethics committee, informed consent was waived because the study involved analysis of existing data without any prospective data collection or direct interaction with participants. Consent to publish was not required, as the study used fully anonymized retrospective data, and the ethics committee granted a waiver of informed consent.

## Conflicts of Interest

The authors declare no conflicts of interest.

## Supporting Information

Additional supporting information can be found online in the Supporting Information section.

## Supporting information


**Supporting Information** Supporting Figure 1. Love plot showing standardized mean differences (SMDs) for baseline covariates before and after propensity score matching. Covariate balance was substantially improved after matching, with all covariates included in the propensity score model achieving an SMD < 0.10 (IPSS‐R: Revised International Prognostic Scoring System). Supporting Table 1: Baseline characteristics of the propensity score–matched cohort according to treatment group.

## Data Availability

The data that support the findings of this study are available from the corresponding author upon reasonable request.
