# Supplementary material for: Real‐World Outcomes of Hypomethylating Agents for Higher‐Risk Myelodysplastic Syndromes: Report From the Nationwide Multicenter Thai MDS Study
Source: Adv Hematol. 2026 Sep 15;2026:2732324. doi: 10.1155/ah/2732324 (PMC13575782; doi:10.1155/ah/2732324)
Supplement: Supplementary file 1 [file AH-2026-2732324-s001.docx]

Supplementary Figure 1. Love plot showing standardized mean differences (SMDs) for baseline covariates before and after propensity score matching. Covariate balance was substantially improved after matching, with all covariates included in the propensity score model achieving an SMD <0.10. (IPSS-R: Revised International Prognostic Scoring System)

Supplementary Table 1: Baseline characteristics of the propensity score–matched cohort according to treatment group.

|  | Best-available treatments  N = 56 | Hypomethylating agents  N=59 | *P* value |
| --- | --- | --- | --- |
| Male (%) | 38 (67.9) | 35 (62.5) | 0.69 |
| Age (median [IQR]) | 70.00 [61.75, 75.25] | 69.00 [62.75, 73.25] | 0.81 |
| Hemoglobin (g/dL) (median [IQR]) | 7.45 [6.72, 8.22] | 7.25 [6.50, 8.65] | 0.84 |
| Absolute neutrophil count (×10³/μL)  (median [IQR]) | 1.06 [0.50, 1.87] | 0.92 [0.57, 1.94] | 0.62 |
| Platelet (×10³/μL) (median [IQR]) | 66 [39, 102] | 93 [48, 138] | 0.05 |
| Bone marrow blast percentage (median [IQR]) | 8.00 [5.00, 15.00] | 10.00 [5.75, 15.00] | 0.46 |
| IPSS-R cytogenetic risk group (%)  Very good Good  Intermediate  Poor  Very poor | 0 (0)  22 (39.3)  15 (26.8)  6 (10.7)  13 (23.2) | 0 (0)  27 (48.2)  11 (19.6)  7 (12.5)  11 (19.6) | 0.71 |
| IPSS-R risk (%)  Intermediate  High  Very high | 7 (12.5)  28 (50.0)  21 (37.5) | 9 (16.1)  26 (46.4)  21 (37.5) | 0.85 |
| IQR: interquartile range, IPSS-R: [revised international prognostic scoring system](https://www.mdcalc.com/calc/3981/revised-international-prognostic-scoring-system-ipss-r-myelodysplastic-syndrome-mds) | | | |
